# Augmented-OvSynch enhances estrus resumption, luteal function, and conception rates in postpartum Zebu crossbred cows under tropical smallholder systems

**DOI:** 10.14202/vetworld.2025.4105-4116

**Published:** 2025-12-27

**Authors:** Suresh Kumar, Megha Pande, Siddhartha Saha, Naimi Chand, Naresh Prasad, Sarmesh Arya, Sumit Mahajan, Umesh Singh, Shantanu Kumar Dubey, Ashok Kumar Mohanty

**Affiliations:** 1 Central Institute for Research on Cattle, Meerut, Uttar Pradesh, India; 2 Agricultural Technology Application Research Institute, Kanpur, Uttar Pradesh, India

**Keywords:** Augmented-OvSynch protocol, estrus synchronization, malondialdehyde, oxidative stress biomarkers, postpartum infertility, progesterone profile, reproductive performance, smallholder dairy systems, total antioxidant capacity, Zebu crossbred cows

## Abstract

**Background and Aim:**

Postpartum infertility remains a major challenge in tropical dairy systems, particularly in Zebu crossbred cows that experience delayed ovarian cyclicity and increased metabolic and environmental stress. Improving early conception is essential to reducing calving intervals and enhancing productivity. This study evaluated the effectiveness of an Augmented-OvSynch protocol incorporating a post-insemination gonadotropin-releasing hormone (GnRH) injection in improving reproductive performance and assessing associated endocrine and oxidative stress dynamics in postpartum Zebu crossbred cows under field conditions.

**Materials and Methods:**

A total of 219 clinically healthy pluriparous Zebu crossbred cows (Holstein-Friesian × Sahiwal), 55–90 days postpartum, were randomly allocated to either a control group receiving the standard OvSynch protocol (n = 82) or a treatment group receiving the Augmented-OvSynch protocol (n = 137), which included an additional GnRH dose on day 5 post-artificial insemination (AI). Estrus response, follicular dynamics, pregnancy rate, and pregnancy loss were monitored through behavioral signs and ultrasonography. Blood samples collected on days 0, 5, 10, and 21 were analyzed for estrogen, progesterone, malondialdehyde (MDA), superoxide dismutase (SOD), and total antioxidant capacity (TAC). Statistical significance was set at p < 0.05.

**Results:**

The Augmented-OvSynch protocol significantly improved estrus expression (83.94%) compared with the control group (37.80%). Pregnancy rate per AI was markedly higher in treated cows (68.61%) than in controls (29.27%). Progesterone concentrations increased significantly from day 5 to day 10 in treated cows, indicating enhanced luteal support and probable accessory corpus luteum formation. Estrogen levels declined more sharply in pregnant cows. Oxidative stress markers were more strongly associated with pregnancy status than protocol type: pregnant cows consistently showed lower MDA and higher TAC levels, whereas SOD exhibited moderate variation without strong treatment effects.

**Conclusion:**

The Augmented-OvSynch protocol substantially improved reproductive outcomes in postpartum Zebu crossbred cows, primarily by enhancing luteal activity and improving synchronization responses. Although oxidative stress markers were influenced more by pregnancy outcome than treatment, their integration provided valuable insight into physiological determinants of fertility. Owing to its simplicity, low-cost, and field suitability, the protocol offers a practical and scalable reproductive management strategy for tropical smallholder dairy systems.

## INTRODUCTION

Establishing early pregnancy following calving is essential to improving reproductive efficiency and sustaining productivity in cattle production systems. Timely insemination during the early postpartum period contributes to shorter calving intervals and supports higher milk yield and overall herd performance [1]. However, in tropical dairy environments, factors such as heat stress, elevated ambient temperatures, high humidity, and endemic infectious pressures negatively influence fertility, making early conception particularly challenging [2, 3]. Native Zebu (*Bos indicus*) cattle further complicate this scenario due to their characteristic delay in resuming ovarian cyclicity after parturition, which hampers achieving the desired 12–14-month calving interval.

Hormonal synchronization protocols in combination with artificial insemination (AI) remain among the most widely used approaches to promote early conception in postpartum cows. These protocols utilize progestins, prostaglandins (e.g., prostaglandin F_2_α [PGF_2_α] and its analogues), or combinations of progestins with estrogens or prostaglandins, and have demonstrated favorable conception rates in Bos taurus breeds. Nonetheless, their effectiveness in Zebu and Zebu crossbred cattle maintained under smallholder field conditions remains insufficiently documented. As dairy production intensifies in tropical smallholder systems, there is a growing demand for cost-effective, simple, and field-adapted hormonal strategies thatenhancereproductive efficiency and improvefarm profitability. The formation of an accessory corpus luteum (ACL) and the associated increase in circulating progesterone have been linked to improved conception outcomes in timed-AI programs [4, 5]. Despite this, the specific benefits of post-insemination ACL induction and the broader applicability of estrus synchronization protocols for postpartum Zebu crossbreds require further investigation. A clearer understanding of the endocrine responses to these interventions, especially in relation to progesterone 94 modulation, may contribute to optimizing fertility under tropical stress conditions.

In addition to hormonal influences, oxidative stress plays a critical role in postpartum fertility. The combined effects of lactational metabolic load, heat stress, and nutritional challenges can intensify oxidative imbalance and impair ovarian function [6, 7]. Increased lipid peroxidation coupled with compromised antioxidant defense has been associated with delayed ovarian rebound and diminished conception rates in *Bos indicus* cows [8, 9]. These interactions highlight the importance of assessing both endocrine and oxidative markers when evaluating reproductive management strategies in tropical dairy systems.

Despite the widespread use of hormonal synchronization protocols for improving fertility in dairy cattle, their effectiveness in Zebu and Zebu crossbred cows under tropical field conditions remains inadequately explored. Most existing evidence on protocols such as OvSynch, progestin–PGF_2_α combinations, and post-AI gonadotropin-releasing hormone (GnRH) administration is derived from *Bos taurus* herds maintained under controlled or intensive management, where nutritional input, climate, and health interventions differ substantially from those in smallholder tropical systems [1–5]. However, Zebu crossbreds exhibit distinct reproductive physiology, including delayed postpartum cyclicity, heightened sensitivity to heat stress, and reduced endocrine responsiveness, all of which can reduce synchronization efficiency [2, 3]. Furthermore, the benefits of inducing an ACL post-insemination, an intervention associated with increased progesterone and improved embryo survival, have not been adequately validated in postpartum *Bos indicus* crossbreds managed under real-world village conditions. Another critical gap lies in the limited integration of oxidative stress biomarkers into fertility research. Tropical dairy cows experience elevated metabolic and thermal stress, predisposing them to oxidative imbalance, which can suppress ovarian function and impair conception [6–9]. Yet, few studies have simultaneously evaluated hormonal dynamics alongside oxidative profiles in synchronized postpartum Zebu crossbred cows. This lack of field-based, physiological, and endocrinological evidence restricts the development of optimized, breed-specific, and climate-appropriate fertility-enhancing protocols.

To address these gaps, the present study aimed to comprehensively evaluate a modified synchronization strategy, the Augmented-OvSynch protocol, designed to enhance conception efficiency in postpartum Zebu crossbred cows under tropical smallholder conditions. Specifically, the study sought to (i) determine the impact of incorporating a day-5 post-AI GnRH injection on estrus expression, ovulation, and conception outcomes relative to the standard OvSynch protocol; (ii) characterize the associated endocrine responses by assessing the temporal patterns of circulating estrogen and progesterone during the early luteal phase; and (iii) investigate the relationship between reproductive outcomes and oxidative stress status by quantifying key biomarkers, including malondialdehyde (MDA), superoxide dismutase (SOD), and total antioxidant capacity (TAC). By integrating reproductive performance metrics with endocrine and oxidative indicators, this study aims to provide a mechanistic and field-relevant understanding of fertility regulation in postpartum Zebu crossbred cows and to identify a practical, low-cost, and farmer-friendly hormonal intervention suitable for tropical dairy systems.

## MATERIALS AND METHODS

### Ethical approval

The present study was conducted in strict accordance with national and institutional guidelines for the care and use of animals in research. All experimental procedures involving animals were reviewed and approved by the Institutional Animal Ethics Committee (IAEC) of the ICAR–Central Institute for Research on Cattle (ICAR-CIRC), Meerut, India, under the project “Livelihood Improvement through Sustainable Dairy Farming using Suitable Interventions” (Project No. OXX03793). Ethical permission was granted under Approval No. CIRC/IAEC/2024/17.

All procedures adhered to the standards and regulations prescribed by the Committee for the Purpose of Control and Supervision of Experiments on Animals, Government of India. Cows were handled by trained personnel, and all interventions, including hormonal injections, ultrasonography, and blood collection, were performed under the supervision of licensed veterinarians to minimize pain, distress, and unnecessary handling.

Animals selected for the study were clinically healthy, and no invasive procedures beyond routine reproductive management were conducted. AI, hormonal treatments, and biological sample collection were performed using aseptic techniques to ensure animal welfare and biosafety. No animal was subjected to feed or water restriction, and all were maintained under their usual smallholder management practices throughout the study period.

Post-procedure monitoring was conducted to ensure therewere no adverse reactions. No animals were harmed, lost, or euthanized as a result of participation in the study. The study complied with the principles of the replacement, reduction, and refinement (3Rs) by utilizing naturally cycling postpartum cows, ensuring an adequate but minimal sample size for statistical power, and employing procedures that refined animal handling to the greatest extent possible.

### Study period and location

The field study was conducted from October 2021 to March 2024 in villages of western Uttar Pradesh (latitude ≈ 28.99°N; longitude ≈ 77.68°E) under the ICAR programs “My Village–My Pride” and “Farmer FIRST.” The region represents a subtropical agro-climatic zone characterized by hot summers and mild winters, with an average annual temperature of 27°C ± 2°C, relative humidity of 68% ± 5%, annual rainfall of approximately 780 mm, and temperature–humidity index of 78 ± 4, indicating moderate to high thermal stress typical of tropical dairy environments.

### Animals

A total of 219 postpartum Zebu crossbred (Holstein-Friesian [HF] × Sahiwal) lactating cows, all clinically healthy and cyclic, were enrolled in this study. Animals were in their second to fifth parity (distribution: 2nd = 45%, 3rd = 38%, ≥4th = 17%) and were recruited 60–90 days post-calving. Each cow was examined rectally to confirm functional cyclicity and a normal reproductive tract, and only animals with resumed estrous activity without reproductive disorders were included. All cows were reared on smallholder, family-operated farms, with each rearing 3–7 cows under semi-confinement (stall-fed) conditions typical of rural households. They were primarily stall-fed with green fodder and dry roughage, locally grown or sourced from agricultural by-products such as wheat straw, maize stover, and sugarcane tops. Routine management included twice-daily hand milking (06:00 h and 17:00 h), natural suckling, and no early weaning.

The mean body condition score (BCS) was 4.2 ± 0.3 (scale 1–5), indicating good physiological status. The average daily milk yield was 10 ± 1.2 L, with milk fat ≥4% and protein ≥3.4%. The availability of feed and fodder was relatively uniform across villages, ensuring a comparable nutritional and environmental background. All cows were maintained under a routine health and vaccination program, including immunization against foot-and-mouth disease, hemorrhagic septicemia, and black quarter, along with regular deworming and tick control measures as per the government veterinary schedule. Animals were clinically screened before enrollment to confirm freedom from mastitis, metabolic, and parasitic disorders.

AI was performed using frozen-thawed semen from genetically evaluated HF × Sahiwal bulls obtained from the ICAR-CIRC Semen Freezing Laboratory, Meerut, India, to minimize sire-related variability. The AI technicians and ultrasound operators were blinded to the treatment groups.

After stratification by village and parity, cows were randomly allocated using a random-number generator into two groups: control (OvSynch; n = 82) and treatment (Augmented-OvSynch; n = 137). Uneven group sizes reflected farmer consent and the availability of eligible animals across villages. Randomization was stratified by village to minimize clustering effects and ensure uniform distribution of parity and BCS between groups. A *post hoc* power analysis (power >0.80; α = 0.05) confirmed that the total sample size (n = 219) was sufficient to detect meaningful differences in pregnancy rates between treatments. For comparative analyses, these groups were retrospectively subdivided into control pregnant (C-P), control non-pregnant (C-NP), treatment non-pregnant (T-NP), and treatment pregnant (T-P) based on pregnancy outcome.

### Reproductive management

**Figure 1:**
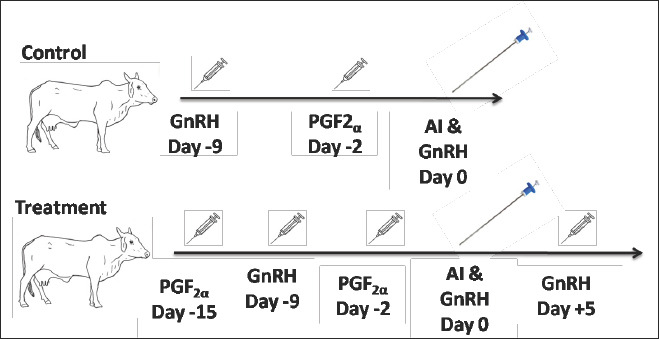
Schematic representation of hormonal treatments used in postpartum Zebu crossbred cows in the control (OvSynch) and treatment (Augmented-OvSynch) groups.

Reproductive management was standardized across both groups (Figure 1), with day 0 defined as the day of AI. Postpartum cows (55–65 days postpartum) were synchronized in the control group using the standard OvSynch protocol (GnRH–PGF_2_α–GnRH). This involved intramuscular (IM) administration of GnRH (Buserelin acetate, Receptal®, MSD, USA; 20 μg in 5 mL) 9 days before AI, PGF_2_α (Cloprostenol sodium, Vetmate®, Intas, India; 500 μg in 2 mL IM) 2 days before AI, and a second GnRH injection (10 μg in 2.5 mL IM) on the day of AI. Fixed-time AI was performed 16–20 h later.

The treatment group received the Augmented-OvSynch protocol (PGF_2_α–GnRH–PGF_2_α–GnRH + post-AI GnRH), beginning with a pre-synchronization dose of PGF_2_α (500 μg in 2 mL IM) 15 days before AI to regress any existing corpus luteum (CL). This was followed by GnRH (20 μg in 5 mL IM) 9 days before AI, a second PGF_2_α injection (500 μg in 2 mL IM) 2 days before AI, and GnRH (10 μg in 2.5 mL IM) on the day of AI, with fixed-time insemination performed 16–20 h later. An additional GnRH injection (10 μg in 2.5 mL IM) was administered 5 days post-AI to induce ovulation and promote ACL formation, thereby supporting luteal function and enhancing fertility outcomes.

### Determination of estrus, ovulation, and pregnancy

Ovarian monitoring was performed using transrectal ultrasonography (Easi-Scan, BCF IMV Technologies, UK) equipped with a 7.5 MHz linear rectal probe at the time of selection and at 24, 48, and 7 days after the second GnRH injection (days +1, +2, and +7 relative to AI, designated as day 0).

Estrus detection was based on both behavioral and US criteria. Behavioral signs, such as standing to be mounted, restlessness, bellowing, sniffing, vulval edema, and clear mucous discharge, were recorded by trained livestock owners following standardized instructions and verified by veterinarians. Two independent observers cross-validated behavioral observations to minimize observer bias, achieving >90% inter-observer agreement (Cohen’s κ = 0.87). Ultrasonographic confirmation of estrus was based on the presence of a pre-ovulatory follicle (≥12 mm), the absence or regression of a CL (≤25 mm), and subsequent ovulation verified by the disappearance of the dominant follicle within 48 h.

Pregnancy diagnosis was performed by transrectal ultrasonography at 35 ± 5 days and reconfirmed at 60 ± 5 days post-AI. The presence of an embryonic vesicle with a viable embryo and heartbeat was considered diagnostic. Non-pregnant cows were re-examined after 60 days to detect late embryonic loss. Diagnostic accuracy was validated against calving records, showing a sensitivity of 96.4% and specificity of 98.2%, consistent with established field validation studies.

### Blood collection, hormone assays, and oxidative stress analysis

Blood samples were aseptically collected from the jugular vein using sterile Vacutainer® (BD, USA) tubes. For hormone assays (estrogen and progesterone), samples were drawn on the day of insemination (day 0) and on days 5, 10, and 21 post-AI using Vacutainer® clot activator tubes (without anticoagulant). Samples for oxidative stress biomarkers were collected on days 0 and 21 using heparinized Vacutainer® tubes (sodium heparin). Samples were immediately placed on ice and centrifuged within 30 min to preserve biomarker stability – plasma at 2,500 × *g* for 10 min and serum at 2,000 × *g* for 20 min at 4°C. Plasma and serum aliquots were stored at −80°C and −20°C, respectively.

Estrogen and progesterone concentrations were determined using commercial enzyme-linked immuno-sorbent assay kits (G Biosciences, USA; estrogen Cat No. ITE050240; 17-hydroxyprogesterone Cat No. ITE050074). Assay validation showed a sensitivity of 10 pg/mL, intra-assay CV <8%, and inter-assay CV <10%. All samples were analyzed in duplicate, and the calibration curves (R_2_ > 0.98) were verified for each batch.

Oxidative stress biomarkers, including MDA (a marker of lipid peroxidation), SOD, and TAC, were quantified using established spectrophotometric methods [10–12]. All reagents were of analytical grade, and quality-control samples were included in every run. Sample handling and assay conditions were standardized for all animals to minimize inter-sample variation.

### Statistical analysis

Data were analyzed using the Statistical Package for the Social Sciences v20.0 (IBM Corp., Chicago, IL, USA). The Shapiro–Wilk test was used to assess the normality of continuous variables; non-normal data were log-transformed. Categorical variables (estrus response, pregnancy rate) were analyzed using the chi-square (χ²) test. Continuous variables (hormones, oxidative markers) were expressed as mean ± standard error of the mean (SEM) and analyzed by repeated-measures analysis of variance, with cow ID as a random factor to account for within-animal correlations. Duncan’s multiple-range test was used for *post hoc* comparisons when significant main effects were detected, and Student’s t-test was applied for pairwise comparisons.

To reduce confounding, the statistical model included parity, BCS, and milk yield as covariates. Significance was set at p < 0.05. Where appropriate, 95 % confidence intervals and effect sizes (Cohen’s d) were calculated to indicate the magnitude of differences. Graphs were created in GraphPad Prism (GraphPad Software Inc., San Diego, CA, USA) with error bars representing mean ± SEM.

## RESULTS

### Estrus response and follicular dynamics

In the treatment group, the number of cows showing estrus was significantly higher than in the control group (83.94% vs. 37.80%). The percentage of cows in true estrus was, however, slightly higher in the control group than in the treatment group (93.54% vs. 90.43%). The size of the dominant follicle was reported to be 14.45±0.24 and 12.48 ± 0.55 mm in the control and treatment groups, respectively. Pregnancy per AI at 35 and 60 ± 5 days after AI and pregnancy loss are presented in Table 1. No adverse reactions or dropouts were observed during treatment.

**Table 1:** Estrus resumption in post-parturient lactating cows subjected to hormonal treatment.

Parameters	Control (n = 82)	Treatment (n = 137)
Cows showing estrus (n)	31	115
Percentage of cows showing estrus (%)	37.80^a^	83.94^b^
Duration of estrous* (h)	21.41 ± 1.03	24.21 ± 1.86
Cows in true estrus (n)	29	104
Percentage of cows showing true estrus (%)	93.54	90.43
Cows in false estrus (n)	2	11
Percentage of cows showing false estrus (%)	6.45	9.56
Number of cows pregnant at 35 ± 5 days	27	98
Number of cows pregnant at 60 ± 5 days	24	94
Pregnancy loss (%)	11.11 (3/27)	4.08 (4/98)
Pregnancy rate (%)	29.27^a^ (24/82)	68.61^b^ (94/137)

Values bearing different superscripts (a, b) in a column differ significantly (p < 0.05).

* Data are presented as mean ± SEM. SEM = Standard error of the mean.

### Effect of hormonal intervention on estrogen levels

Blood estrogen levels during the modified hormonal protocol showed distinct temporal changes across the groups (Table 2). On day 0, estrogen concentrations were significantly higher (p < 0.05) in the treatment groups than in the control groups, particularly among pregnant cows. In pregnant cows (both 35 ± 5 and 60 ± 5 days), estrogen levels decreased progressively from day 0 to 10, reflecting the expected postovulatory dynamics. Notably, estrogen levels remained low by day 21 in the pregnant groups. In contrast, non-pregnant cows in the treatment group showed a significant decrease in estrogen levels by day 10 (p < 0.05), but an unexpected increase was observed again by day 21. Overall, treatment and pregnancy outcome influenced the estrogen pattern, with a clearer decline in successful pregnancies.

**Table 2:** Effect of hormonal intervention on estrogen levels in blood (pg/mL) in post-parturient lactating cows.

Parameters	n	Day 0	Day 5	Day 10	Day 21
Control (non-pregnant)	55	1.59 ± 0.07^Aa^	1.26 ± 0.07^a^	0.85 ± 0.02^Ab^	1.25 ± 0.02^Aa^
Control (pregnant, 35 ± 5 days)	27	1.64 ± 0.02^ABa^	1.18 ± 0.09^a^	0.89 ± 1.023^Ab^	0.89 ± 0.03^Bb^
Control (pregnant, 60 ± 5 days)	24	1.78 ± 0.06^ABa^	1.05 ± 0.05^a^	0.98 ± 0.07^Ab^	0.68 ± 0.07^Bb^
Treatment (non-pregnant)	39	1.86 ± 0.05^Ba^	1.14 ± 0.02^a^	0.71 ± 0.01^Bb^	1.84 ± 0.04^Ab^
Treatment (pregnant, 35 ± 5 days)	98	2.05 ± 0.01^Ba^	1.01 ± 0.00^b^	0.61 ± 0.05^Bc^	0.68 ± 0.07^Bc^
Treatment (pregnant, 60 ± 5 days)	94	2.41 ± 0.07B^a^	1.87 ± 0.10^b^	0.68 ± 0.07^Bc^	0.69 ± 0.03^Bc^

Values bearing different superscripts (a, b) in a row and column (A, B) differ significantly (p < 0.05). Data are presented as mean ± SEM. SEM = Standard error of the mean.

### Effect of hormonal intervention on progesterone levels

The effect of hormonal intervention on blood progesterone levels (ng/mL) in postpartum lactating cows is presented in Table 3. Both the treatment and control groups showed a statistically significant (p < 0.05) increase in blood progesterone levels from day 0 to day 5. Notably, from day 5 to day 10, the treatment group exhibited a significantly greater increase in progesterone levels than the control group. In non-pregnant cows, progesterone levels in the treatment group increased from 2.24 ± 0.05 ng/mL to 6.71 ± 0.11 ng/mL, whereas those in the control group showed a more modest increase from 2.26 ± 0.05 ng/mL to 4.18 ± 0.02 ng/mL.

**Table 3:** Effect of hormonal intervention on blood progesterone levels (ng/mL) in post-parturient lactating cows.

Parameters	n	Day 0	Day 5	Day 10	Day 21
Control (non-pregnant)	55	0.41 ± 0.06^a^	2.26 ± 0.05^b^	4.18 ±.02^Ac^	0.75 ± 0.06^Aa^
Control (pregnant, 35 ± 5 days)	27	0.45 ± 0.05^a^	2.18 ± 0.04^b^	5.07 ± 1.023^Ac^	5.56 ± 0.11^Bc^
Control (pregnant, 60 ± 5 days)	24	0.51 ± 0.02^a^	2.45 ± 0.07^b^	5.98 ± 0.07^Ac^	6.65 ± 0.21 ^Bd^
Treatment (non-pregnant)	39	0.41 ± 0.07^a^	2.24 ± 0.05^b^	6.71 ± 0.11^Bc^	0.95 ± 0.10^Ac^
Treatment (pregnant, 35 ± 5 days)	98	0.42 ± 0.06^a^	2.26 ± 0.03^b^	6.41 ± 0.09^Bc^	6.82 ± 0.12 ^Bc^
Treatment (pregnant, 60 ± 5 days)	94	0.47 ± 0.05^a^	2.57 ± 0.08^b^	6.58 ± 0.10^Bc^	6.97 ± 0.10 ^Bc^

Values bearing different superscripts (a, b) in a row and column (A, B) differ significantly (p < 0.05). Data are presented as mean ± SEM. SEM = Standard error of the mean.

In pregnant cows, the treatment group (both 35 ± 5 days and 60 ± 5 days) showed significantly higher progesterone levels on day 10 than the control group (6.41 ± 0.09 ng/mL and 6.58 ± 0.10 ng/mL, respectively, versus 5.07 ± 1.02 ng/mL and 5.98 ± 0.07 ng/mL in controls). These significant changes persisted through day 21, with progesterone levels remaining consistently higher in the treatment group than in the control group.

### Effect of hormonal intervention on antioxidant profile

**Figure 2:**
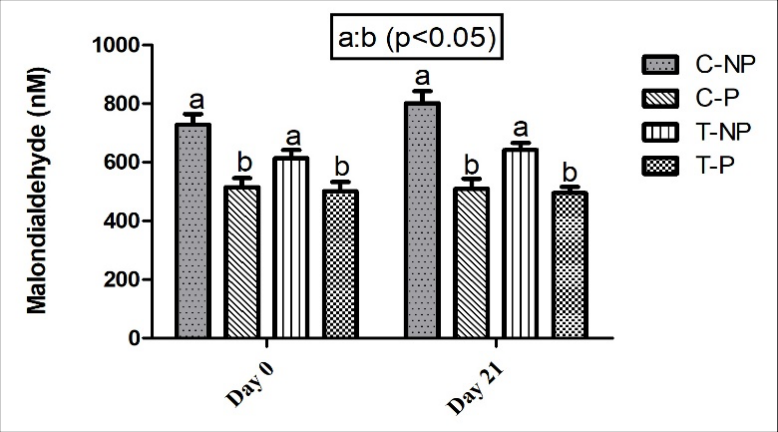
Effect of hormonal intervention on plasma malondialdehyde concentration (nM/mL) in postpartum lactating Zebu crossbred cows. Groups: C-NP = Control non-pregnant, C-P = Control pregnant, T-NP = Treatment non-pregnant, and T-P = Treatment pregnant. Values represent mean ± standard error of the mean on days 0 and 21 after artificial insemination. The bars with different superscripts (a, b) within each time point differ significantly (p < 0.05).

Plasma MDA levels, representing lipid peroxidation, were significantly higher (p < 0.05) in non-pregnant cows than in pregnant cows in both treatment and control groups (Figure 2). No significant difference in MDA levels was observed between the treatment and control groups within the same pregnancy status. This suggests that the oxidative stress status, as reflected by MDA, was more influenced by pregnancy outcome than by the hormonal treatment protocol.

**Figure 3:**
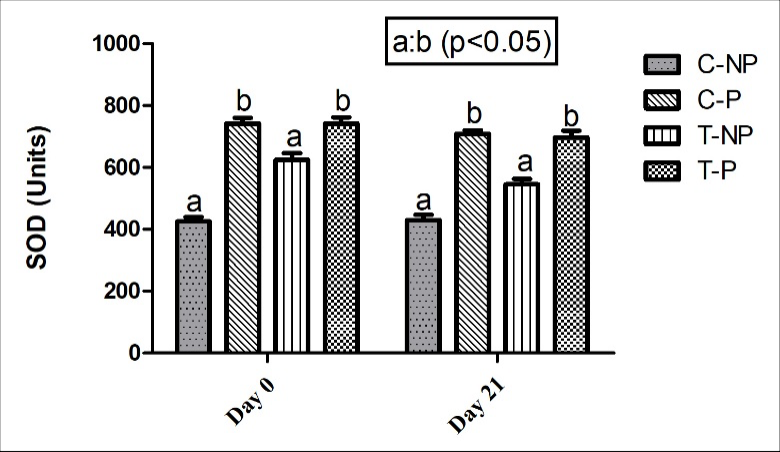
Effect of hormonal intervention on plasma superoxide dismutase activity (U/mL) in postpartum lactating *Zebu* crossbred cows. Groups: C-NP = Control non-pregnant, C-P = Control pregnant, T-NP = Treatment non-pregnant, and T-P = Treatment pregnant. Values represent mean ± standard error of the mean at 35 ± 5 days post-artificial insemination. The bars with different superscripts (a, b) within each time point differ significantly (p < 0.05).

SOD levels were generally higher in the treatment groups than in the controls on day 0, particularly in non-pregnant cows (Figure 3). However, by day 21, SOD levels showed a non-significant decrease in both pregnant and non-pregnant animals within the treatment group (p > 0.05). No consistent treatment-related pattern was observed across time points. The primary difference was again between pregnant and non-pregnant cows, rather than between the treatment and control groups.

**Figure 4:**
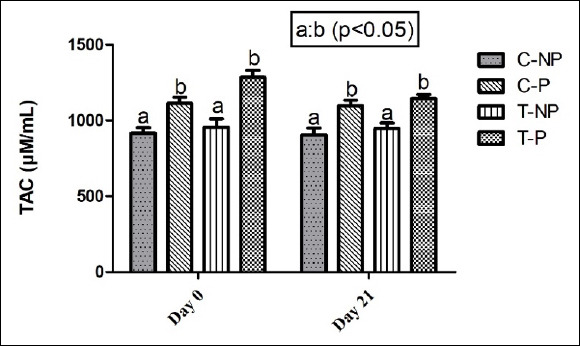
Effect of hormonal intervention on plasma total antioxidant capacity (nM/mL) in *Zebu* crossbred cows that are lactating postpartum. Groups: C-NP = Control non-pregnant, C-P = Control pregnant, T-NP = Treatment non-pregnant, and T-P = Treatment pregnant. Values represent mean ± standard error of the mean at 35 ± 5 days post-artificial insemination. The bars with different superscripts (a, b) within each time point differ significantly (p < 0.05).

As shown in Figure 4, TAC was significantly higher (p < 0.05) in pregnant cows than in non-pregnant cows on days 0 and 21 in both the treatment and control groups. Within-group changes over time were minor, and no significant treatment effect was observed. Thus, TAC levels reflected a pregnancy-associated antioxidant advantage, independent of treatment.

## DISCUSSION

### Postpartum reproductive challenges in Zebu crossbreeds

Postpartum anestrus remains a persistent reproductive challenge in *Bos indicus* cattle, resulting in extended calving intervals and reduced fertility [6, 7, 9]. In tropical dairy systems, timely resumption of ovarian cyclicity is crucial for sustaining productivity. Although hormonal synchronization protocols have shown success in *Bos taurus* and buffaloes under intensive management [13, 14], their direct adaptation to *Zebu* and crossbred cows maintained by smallholders under nutritional and thermal stress is less explored. This field-based study assessed a modified Augmented-OvSynch protocol designed to enhance early luteal function and conception efficiency under these conditions.

### Efficacy of the Augmented-OvSynch protocol

The inclusion of a third GnRH injection on day 5 post-AI in the Augmented-OvSynch protocol was intended to promote accessory CL formation and elevate plasma progesterone during early luteal development. This modification significantly improved estrus response (83.94%) and conception rate (68.61%) compared with the standard OvSynch protocol. These results are consistent with those of previous studies by by García-Guerra *et et al*. [15] and Borş *et al*. [16], which showed that post-AI GnRH can stimulate luteal function, resulting in a hormonal milieu favorable for embryo survival.

The luteotropic effect of GnRH is mediated by an leuitinizing hormone (LH) surge that enhances luteal cell differentiation and progesterone synthesis [17–19]. The timing of the third dose of GnRH (day 5) was chosen to coincide with the early luteal phase, when the dominant follicle remains responsive to LH. Progesterone concentrations in the present study rose markedly from day 5 to day 10, with a greater increase in treated cows (6.71 ± 0.11 ng/mL) than controls (4.18 ± 0.02 ng/mL), confirming improved luteal activity. Although pregnancy loss was numerically lower in the treatment group, the difference was not statistically significant, suggesting that elevated progesterone alone may not fully prevent embryonic mortality, which is often linked to inadequate anti-luteolytic signaling [20–22]. Refinement of post-AI hormone timing or integration with supportive therapies could further reduce early losses.

### Hormonal and endocrine dynamics

The observed trends in estradiol and progesterone were consistent with previous findings in *Bos indicus* and beef cows [23, 24]. Pregnant cows in the treatment group exhibited a sharper decline in estradiol by day 10, possibly reflecting altered follicular turnover after GnRH-induced ovulation. Both progesterone and estradiol are essential for endometrial preparation, embryo implantation, and pregnancy maintenance [25, 26]. Variations in these hormone patterns across studies likely stem from breed-specific endocrine responsiveness and environmental influences.

### Role of oxidative stress in fertility

Oxidative stress is a major determinant of reproductive efficiency in dairy cattle [27]. Non-pregnant cows exhibited significantly higher MDA levels, indicating greater lipid peroxidation, whereas pregnant cows had higher TAC and SOD activity. These findings are consistent with earlier reports linking oxidative imbalance to impaired luteal function and reduced conception in cattle and buffaloes [28–31]. Elevated MDA levels have been associated with disrupted estrogen–progesterone ratios, accelerated luteolysis, and repeat breeding [32], whereas improved antioxidant defense supports follicular integrity and embryo survival [33, 34].

Both estrogen and progesterone modulate antioxidant defense mechanisms [35–37]. Estrogen exerts mild membrane-stabilizing and antioxidative effects [38], whereas progesterone exerts limited direct antioxidant activity [39]. The association between higher antioxidant status and successful conception observed in this study indicates a positive, though not necessarily causal, relationship. As oxidative balance is influenced by nutrition, the environment, and health, further controlled studies are warranted to delineate its mechanistic role.

### Integration of antioxidants with hormone synchronization

No exogenous antioxidants (vitamin E, selenium, or β-carotene) were administered, ensuring that the observed differences reflected physiological variation. However, incorporating antioxidants could complement hormonal synchronization by reducing oxidative damage and supporting luteal function. Previous studies by Xiao *et al*. [40] and Dhami *et al*. [41] have reported fertility challenges in Zebu and crossbred cows in tropical field systems. Hormonal protocols, such as controlled internal drug release, OvSynch, and calf-removal methods, have improved service intervals and conception [42–44]; however, few oxidative markers have been assessed.

This study provides novel insights by linking endocrine responses with oxidative stress markers, offering a more integrated physiological understanding of synchronization outcomes. Studies in buffaloes and crossbred cattle have demonstrated enhanced follicular dynamics and conception when antioxidants are combined with hormonal regimens [45, 46]. Vitamin E and selenium supplementation have been shown to improve antioxidant status and reproductive efficiency by reducing services per conception and days open [47, 48]. Integrating these nutritional strategies, especially vitamin E, selenium, or herbal antioxidants [49, 50], may further enhance conception rates in tropical environments.

### Practical applicability and farmer compliance

The Augmented-OvSynch protocol was designed for use under smallholder conditions, using commonly available hormonal preparations and requiring only one additional GnRH injection. The extra cost (approximately ₹ 80–160 per cow for a 2.5-ml GnRH injection; USD 0.96–1.92) and a single additional farm visit were minimal compared with the improvement in conception rate and reduced calving interval.. Farmer compliance during the trial was high because the protocol involved familiar injections (GnRH and PGF_2_α) and minimal handling. This practicality and favorable cost–benefit ratio make it suitable for large-scale adoption through AI technicians and veterinary extension services.

### Limitations and prospects

The multi-farm field design introduced unavoidable variation in management, feeding, and microclimatic factors, although bias was minimized by randomization. The sample size (n = 219) provided sufficient power (0.80) to detect treatment effects but limited subgroup analyses. Nutritional antioxidant intake was not quantified, and inseminators were not completely blinded. Despite these constraints, the study’s real-world context strengthens its practical relevance. Long-term reproductive parameters, such as calving interval, days open, lifetime milk yield, and conception efficiency across lactations, were not assessed and merit future longitudinal evaluation.

Future research should focus on optimizing GnRH timing and exploring synergistic hormonal-nutritional approaches, especially vitamin E, selenium, and natural antioxidants, to enhance reproductive resilience in Zebu crossbreds under tropical stress conditions.

## CONCLUSION

The present field-based investigation demonstrates that the Augmented-OvSynch protocol substantially improves reproductive performance in postpartum Zebu crossbred cows managed under tropical smallholder conditions. The addition of a day-5 post-insemination GnRH injection markedly enhanced estrus expression (83.94% vs. 37.80%) and pregnancy rate (68.61% vs. 29.27%) compared with the standard OvSynch protocol, confirming the effectiveness of inducing accessory CL formation and strengthening luteal support during early gestation. Treated cows exhibited a more favorable progesterone profile, with significantly higher concentrations from days 5 to 21, reflecting improved luteal activity and a hormonal environment conducive to embryo survival. Although oxidative stress markers were more strongly influenced by pregnancy status than protocol type, pregnant cows consistently demonstrated lower MDA levels and higher TAC, underscoring the physiological linkage between redox balance and successful conception.

A major strength of this study is its real-world applicability: the trial was conducted across multiple villages, using farmer-managed cows, locally available hormonal preparations, and minimal additional inputs. The protocol required only a single extra GnRH injection, incurred negligible added cost, and achieved high farmer compliance, making it a practical, scalable intervention suitable for large-scale reproductive management programs. Additionally, the integrated evaluation of endocrine responses and oxidative stress biomarkers provided a broader physiological insight, highlighting interactions that are seldom assessed together in field conditions.

In conclusion, the Augmented-OvSynch protocol represents a simple, cost-effective, and biologically sound strategy for enhancing fertility in postpartum Zebu crossbred cows in tropical environments. Its ability to improve estrus synchronization, strengthen luteal dynamics, and elevate conception rates underscores its value for improving herd productivity in smallholder dairy systems. Future studies should refine hormone timing and explore synergistic nutritional strategies, particularly antioxidant supplementation, to further enhance reproductive resilience and long-term fertility outcomes.

## DATA AVAILABILITY

The data supporting the results of this study are available from the corresponding author upon reasonable request.

## AUTHORS’ CONTRIBUTIONS

SK and MP: Conceptualization and methodology.NP and US: Software. SK, MP, and NP: Validated the study. SK: Formal analysis and funding acquisition. SK, SA, MP, and SS: Investigated the study. AKM, NC, ASS, and SM: Resources. NC: Data curation.MP: Writing, original draft preparation, and visualization. SK, MP, and ASS: Wrote, reviewed, and edited the study. AKM: Supervised the study. SK and SKD: Project administration. All authors have read and approved the final version of the manuscript.
